# Supplementary material for: Epistemic trust towards teacher questionnaire: Development and preliminary validation
Source: PLoS One. 2025 Sep 15;20(9):e0331398. doi: 10.1371/journal.pone.0331398 (PMC12435716; doi:10.1371/journal.pone.0331398)
Supplement: S1 Questionnaire — (DOCX) [file pone.0331398.s001.docx]

**S 1 Questionnaire. The epistemic trust towards teacher questionnaire**

**Please work through the following 18 statements regarding your teacher. For each statement, choose a number between 1 (Strongly Disagree) and 5 (Strongly Agree) to say how much you disagree or agree with the statement, and write it beside the statement. Thank you.**

**Use the following scale from 1 to 5:**

Strongly 1 2 3 4 5 Strongly

disagree agree

1. __ Sometimes we laugh and joke together with this teacher **(original item ETQ35)**
2. __ This teacher does not pay attention to me. **(original item ETQ5)**
3. __ Things I learn from this teacher are useful in real life. **(original item ETQ23)**
4. __ This teacher often smiles at me. **(original item ETQ1)**
5. __ This teacher never calls me by my name. **(original item ETQ43)**
6. __ Some things I learned from this teacher were useful outside the class **(original item ETQ37)**
7. __ I trust this teacher. **(original item ETQ22)**
8. __ I often feel this teacher does not listen to me. **(original item ETQ12)**
9. __ I don't see how to apply what we learn in this teacher's class outside of school. **(original item ETQ32*)**
10. __ This teacher notices when I don't understand something. **(original item ETQ39)**
11. __ This teacher is not interested in what I have to say. **(original item ETQ18)**
12. __ What I learn from this teacher is going to be useful in other settings. **(original item ETQ4)**
13. __ This teacher understands me. **(original item ETQ49)**
14. __ This teacher often does not say hello to me, even when they see me. **(original item ETQ29)**
15. __ I feel what I am learning from this teacher is useless. **(original item ETQ47*)**
16. __ I feel that my success is important to this teacher. **(original item ETQ51)**
17. __ I do not trust this teacher. **(original item ETQ40)**
18. __ I often find things I learned from this teacher useful in other subjects. **(original item ETQ50)**
