# Supplementary material for: Epistemic trust towards teacher questionnaire: Development and preliminary validation
Source: PLoS One. 2025 Sep 15;20(9):e0331398. doi: 10.1371/journal.pone.0331398 (PMC12435716; doi:10.1371/journal.pone.0331398)
Supplement: S4 Questionnaire — (DOCX) [file pone.0331398.s003.docx]

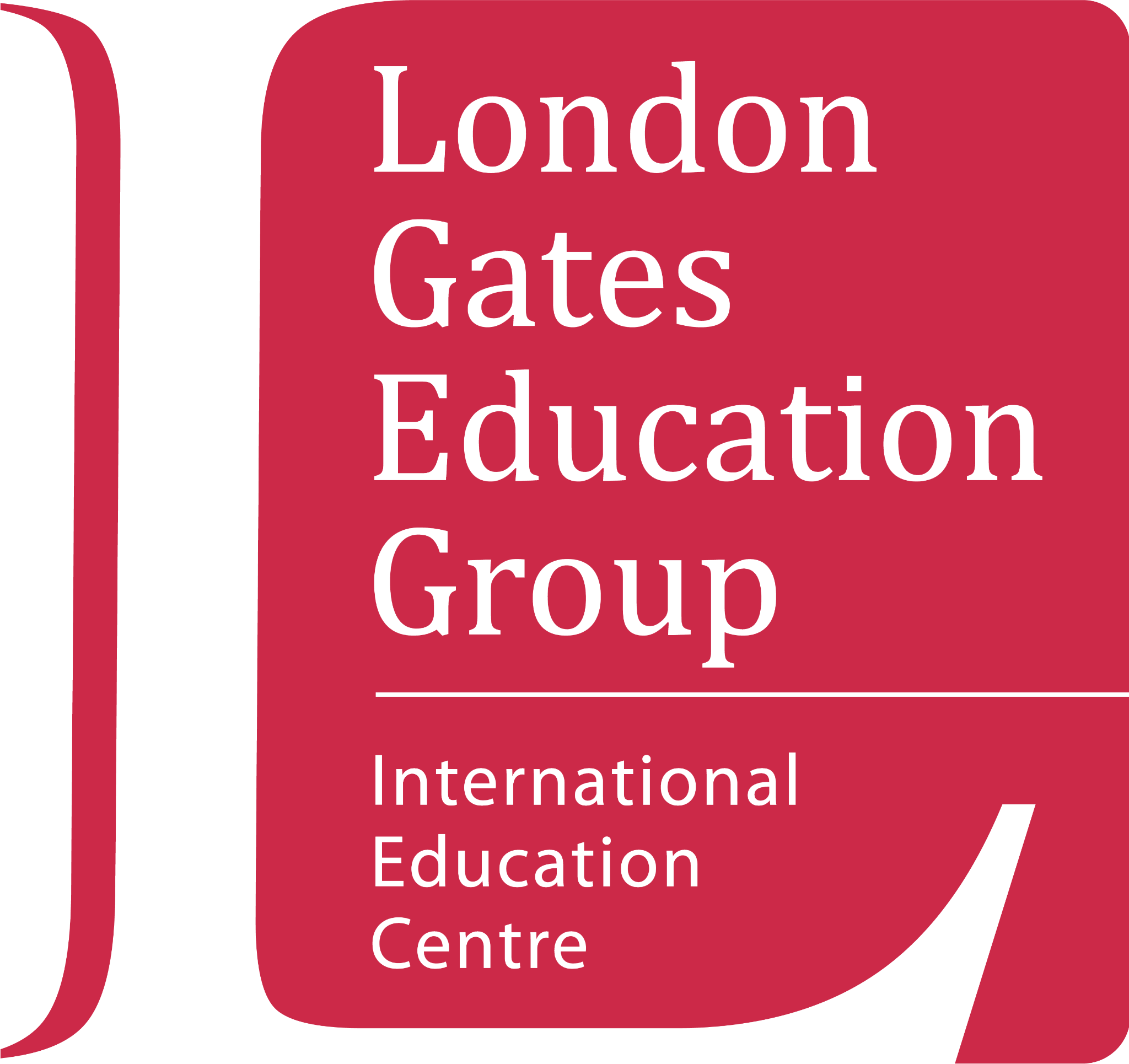


**S 2. Ethical approval letter from the Ethical Review Board**

Minimal Risk Research

London Gates Education Group

Ethical Review Board (06/03/2021)

**Submission Protocol Number:**

**Title:**

**Principal Investigator:**

**Point-of-contact:**

**ERB Stuff Reviewers:**

53485

Epistemic trust towards teacher questionnaire: Development and preliminary validation

Alex Desatnik

Maxim Yakubovskiy

Alex Balakirev, Nina Symonian, Mary Vinograd

The London Gates Education Group Ethical Review Board conducted a review of the above-referenced application. The study was determined to meet the criteria for exempt human subjects in accordance with the following category(ies) as defined under 45 CFR 46:

(2)(ii) Tests, surveys, interviews, or observation (low risk).

If this study falls under VA regulations, you must get final approval from the VA Research & Development Committee prior to starting research activities.

You have identified the following financial sources to support the research activities in this ERB application:

None.

If this information is incorrect, please submit a change to modify your application as appropriate.

If you have general questions, please contact the Minimal Risk Research ERB at +371 66 010 108. For questions related to this submission, contact the assigned staff reviewers.
