# Supplementary material for: Epistemic trust towards teacher questionnaire: Development and preliminary validation
Source: PLoS One. 2025 Sep 15;20(9):e0331398. doi: 10.1371/journal.pone.0331398 (PMC12435716; doi:10.1371/journal.pone.0331398)
Supplement: Attachment — Submitted filename: Response to Reviewers.docx [file pone.0331398.s005.docx]

Dear Dr. Karimi and Reviewers,

We would like to thank you and the reviewers for the thoughtful and constructive feedback on our manuscript, titled “Epistemic trust towards teacher questionnaire: Development and preliminary validation.” We greatly appreciate the opportunity to revise our submission and are pleased that the reviewers found the manuscript to be of high quality, with a well-justified design, accurate data analysis, and a convincing discussion. We are particularly grateful for the reviewer’s encouraging comments regarding the theoretical potential of epistemic trust in educational contexts. We are also thankful for the reviewers’ recognition of the study’s theoretical grounding, methodological rigor, and practical relevance.

Below, we provide point-by-point responses to all comments, describing the revisions made in response.

**Reviewer #1 Comment:**
“*It would be very necessary to provide more information about the implications of epistemic trust in education. For example, the authors may refer to more potentially-related correlates of epistemic trust in learning*.”

**Response:**
We thank the reviewer for this thoughtful and constructive suggestion. In response, we have expanded the Discussion section to offer a deeper theoretical interpretation of the “Generalization” subscale and its relevance for educational practice. We highlight how this dimension reflects students’ perception of the real-world applicability of knowledge and propose implications for teacher communication strategies, classroom design, and teacher training. We also briefly discuss how this measure can be used to guide interventions or inform educational policy. This addition is now included on page 30 of the revised manuscript.

Additionally, we have addressed the minor revisions suggested regarding grammar and APA style formatting to ensure clarity and adherence to publication standards.

**Reviewer #2 Comment (Abstract):**
“*The abstract should include model fit indices from the CFA and reliability coefficients. Additionally, phrases such as “relatively distinct and theoretically coherent” should be replaced with more precise language.*”

**Response:**
We thank the reviewer for these insightful suggestions. We have revised the abstract to enhance clarity and align it with psychometric reporting standards. Specifically, we now report CFA model fit indices (χ²/df, CFI, TLI, RMSEA) and Cronbach’s alpha coefficients for each subscale. We have also replaced vague phrasing with more precise language describing the discriminant validity and theoretical alignment of the three factors. The revised abstract appears on page 2 of the manuscript.

**Reviewer #2 Comment (Introduction):**
“*While the introduction is strong, some theoretical content (particularly on the evolutionary basis of learning and generalization) is repeated and could be condensed. Methodological content (e.g., item development and hypotheses) may be better suited for the Methods section. Transitions and grammar should also be improved*.”

**Response:**
We appreciate this valuable feedback. The introduction has been revised to improve readability and logical flow. We have removed repetitive content, streamlined the discussion of evolutionary theory and generalization, and relocated methodological details (e.g., scale development procedures, hypotheses) to the Methods section on pages 7-8 of the Manuscript. We have also refined transitions and edited for clarity and conciseness throughout the section.

### **Reviewer #2 Comment (Methods – Clarity and Structure):**

“*The Methods section is densely written and would benefit from subheadings (e.g., “Item Development,” “Participants and Procedures,” etc.). Long sentences (e.g., lines 185–193) should be simplified.*”

**Response:**
 Thank you for this constructive suggestion. We have restructured the Methods section to include clear subheadings: “Development of the epistemic trust towards teachers questionnaire,” “Study hypothesis,” “The development of the scale,” “Participants and Procedures,” and “Other Measures.” We have also revised lengthy sentences for clarity and readability, as advised.

### **Reviewer #2 Comment (Methods – Item Reduction Procedure):** “*The criteria for item rejection are not fully explained. Please clarify how items were selected for CFA beyond expert input and whether inter-rater agreement was assessed*.”

**Response:**
We thank the reviewer for pointing this out. We have expanded the manuscript to clarify our item reduction procedure. Specifically, items were evaluated by seven international experts based on clarity, relevance, and redundancy. Items were retained if at least five experts rated them as highly relevant and non-redundant. While we did not compute formal inter-rater agreement statistics, a structured consensus procedure modeled on the Delphi method was used, incorporating independent review and iterative feedback. These details have been added to the “The development of the scale” section on page 10.

**Reviewer #2 Comment (Sampling and Demographics):**
“*The sample is highly homogeneous (urban, upper-middle-class). This limitation should be acknowledged, as it affects generalizability*.”

**Response:**
 We fully agree with this critical point. We have revised the Limitations section (pages 28–30) to explicitly acknowledge the socioeconomic and geographic homogeneity of our sample. We discuss how this limitation affects the generalizability and interpretation of findings across more diverse or cross-cultural populations. We also note the need for future validation of the scale in broader and more diverse samples.

### **Reviewer #2 Comment (Ethical Procedures):** “*The ethical procedures are adequately described, but it would help to state explicitly that data were anonymous and voluntary*.”

**Response:**
 We appreciate this suggestion. We have now explicitly stated that participation was both anonymous and voluntary, in order to reinforce adherence to ethical standards. This clarification appears on pages 10–11 of the revised manuscript.

### **Reviewer #2 Comment (Discussion – Overuse of General Statements):** “*Phrases such as “seem to provide preliminary support” or “may be promising” are overused. Please use more assertive language where justified by strong data.*”

**Response:**
Thank you for this helpful observation. We have revised the Discussion section to reduce hedging language and adopt more confident phrasing where the data are statistically robust. For example, we now state that the findings “a similar pattern was observed,” “these findings indicate,” “study provides encouraging evidence” rather than “seem to provide,” and have made similar adjustments throughout the paper.

### **Reviewer #2 Comment (Discussion – Limited Depth in Interpretation):**

“*The discussion would benefit from deeper theoretical interpretation—particularly of the “Generalization” subscale—and its implications for educational practice and policy.*”

**Response:**
We thank the reviewer for this important recommendation. We have expanded the Discussion section to offer a deeper interpretation of the “Generalization” subscale and its practical relevance. We propose that this dimension reflects students’ perception of the real-world applicability of knowledge, which may inform strategies in classroom communication, instructional design, and teacher training. We also comment on how this construct could guide targeted interventions and educational policy. This revision is included on page 30 of the revised manuscript.

### **Reviewer #2 Comment (Discussion – Acknowledgment of Limitations):** “*Limitations are generally acknowledged, but sampling limitations should be more explicitly tied to generalizability.*”

**Response:**
 We agree and have revised the Limitations section (pages 28–30) to more clearly connect sample characteristics — especially the upper-middle-class, urban background of participants — to constraints on generalizability. We emphasize the importance of future studies validating the ETT scale in more diverse socioeconomic and cultural settings.

Once again, we are grateful for the opportunity to revise the manuscript and for the reviewers’ positive evaluation of our work. We sincerely thank the Academic Editor and both Reviewers once again for their detailed and insightful comments. These suggestions significantly improved the clarity, depth, and impact of the manuscript.

Sincerely,
Max Yakubovskiy

On behalf of all co-authors

Department of Educational Policy Studies,

School of Education,

University of Wisconsin-Madison, WI, USA

yakubovskiy@wisc.edu
